# The Role of C-X-C Chemokine Receptor Type 4 (CXCR4) in Cell Adherence and Spheroid Formation of Human Ewing’s Sarcoma Cells under Simulated Microgravity

**DOI:** 10.3390/ijms20236073

**Published:** 2019-12-02

**Authors:** Alexander Romswinkel, Manfred Infanger, Carlo Dietz, Florian Strube, Armin Kraus

**Affiliations:** Department of Plastic, Aesthetic and Hand Surgery, Otto-von-Guericke-University, Leipziger Strasse 44, D-39120 Magdeburg, Germany; alexander.romswinkel@st.ovgu.de (A.R.); manfred.infanger@med.ovgu.de (M.I.); carlo.dietz@st.ovgu.de (C.D.); florian.strube@med.ovgu.de (F.S.)

**Keywords:** cell adhesion, cytoskeleton, cell culture techniques, hypogravity, microgravity, neoplasms, sarcoma, CXCR4, plerixafor

## Abstract

We studied the behavior of Ewing’s Sarcoma cells of the line A673 under simulated microgravity (s-µg). These cells express two prominent markers—the oncogene EWS/FLI1 and the chemokine receptor CXCR4, which is used as a target of treatment in several types of cancer. The cells were exposed to s-µg in a random-positioning machine (RPM) for 24 h in the absence and presence of the CXCR4 inhibitor AMD3100. Then, their morphology and cytoskeleton were examined. The expression of selected mutually interacting genes was measured by qRT-PCR and protein accumulation was determined by western blotting. After 24 h incubation on the RPM, a splitting of the A673 cell population in adherent and spheroid cells was observed. Compared to 1 g control cells, *EWS/FLI1* was significantly upregulated in the adherent cells and in the spheroids, while *CXCR4* and *CD44* expression were significantly enhanced in spheroids only. Transcription of *CAV-1* was upregulated and *DKK2* and *VEGF-A* were down-regulated in both, adherent in spheroid cells, respectively. Regarding, protein accumulation EWS/FLI1 was enhanced in adherent cells only, but CD44 decreased in spheroids and adherent cells. Inhibition of CXCR4 did not change spheroid count, or structure. Under s-µg, the tumor marker EWS/FLI1 is intensified, while targeting CXCR4, which influences adhesion proteins, did not affect spheroid formation.

## 1. Introduction

Ewing’s sarcoma (ES) is the s most common bone tumor in adolescents and young adults, accounting for approximately 3–5% of all pediatric tumors [1,2]. Despite major advances in therapy of patients with localized disease over the last decades [3,4,5], outcome of patients with clinically metastasized or relapsed disease still remains relatively unchanged and poor, with a five-year survival rate of 20–30% [4,6]. Consequently, there is still a substantial need to better understand the process of metastasis formation and cell adherence. Further understanding of these mechanisms appears crucial to provide more effective therapeutic schemes to tackle this cancer. It has repetitively been shown that common conventional 2D cell-culture models have their limitations, mainly due to missing 3D cell–cell and cell–extracellular matrix interactions, which results in more unrealistic proliferation, differentiation, gene expression, vitality, responsiveness to stimuli like chemotherapeutics, and several other cellular functions [7,8,9,10]. In fact it has already been shown for ES itself that multicellular tumor spheroids (MCTS) resemble in vivo tumors much more with respect to morphology, kinase activation, cell–cell junctions and proliferation than their two-dimensional counterparts [11,12]. An elegant way to induce 3D growth in form of MCTS is by the use of microgravity. Research has shown that spaceflights do not only have profound effects on human organisms like, for example, changes in blood pressure [13] or bone mineralization [14], but also on cell cultures. For numerous cell types such as endothelial cells [15,16,17,18,19], which form vessel-like tubes, tenocytes [20], chondrocytes [21], or cancer cells like breast cancer cells [20,21,22], which even form duct-like structures, or thyroid cancer cells [17,22,23,24] detachment of previously firmly attached cells growing in regular monolayer cultures and subsequent aggregation in the supernatant to form three-dimensional structures (spheroids) have been reported. Resemblance of these spheroids even to organoid structures has been reported [15,25], which indicates that s-µg induced spheroids possess a higher level of architecture, and therefore underlying gene expression than one would expect from random three-dimensional aggregates. The phenomenon was observed in real microgravity (r-µg) during space flights [17,26,27] and parabolic flights [21,28], but also in simulated microgravity (s-µg) created in ground-based devices like the random positioning machine (RPM) [29,30,31,32,33]. This way we possess an easy-to-use, scaffold-free method to generate multicellular tumor spheroids that can be used to study underlying cancer biology or for drug testing, penetration or radiation experiments [34,35,36,37,38] due to the characteristic physicochemical gradients and 3D interactions in spheroids, which are similar to those in micrometastases or avascular tumors [8,34,38,39,40,41,42,43]. Moreover, simulated microgravity via RPM involves unique changes in cellular adhesion patterns: The loss of adherence to a cell culture flask of a fraction of formerly firmly attached cells and their reassembly to MCTS thereby switching from two-dimensional adherence to a polystyrene surface to intercellular three-dimensional adherence without undergoing anoikis, while another fraction remains attached under the same circumstances, makes it a particularly intriguing in vitro cancer model. Such a model could help identify molecular switches of adhesion pattern alterations and gain valuable insights in the dynamics of ES cell aggregation and –adherence, which could be of value not only to microgravity, but to cancer researchers in general. In fact, for several cancer entities changes of phenotype mostly towards a less aggressive one under s-µg have been found [44,45,46]. Hence, it is conceivable that studying underlying alterations and pathways could ultimately help to provide new targets to focus on in clinical cancer research, possibly by triggering these mechanisms through ways other than microgravity. Keeping in mind that ES is notoriously known for early metastasis and lack of targeted treatment options, its relevance from a clinical point of view led us to decide to shed light on the impact of s-µg on ES cell adherence [3,5,6]. Several markers have been shown to be associated with ES cell adhesion and metastasis: The translocation protein EWS/FLI1 has been shown to reduce ES cell adhesion in vitro [47] and is speculated to be involved in a functional antagonism with ZEB2 keeping ES cells in a undifferentiated, highly malignant state [48], while the chemokine receptor family (CXCR4, CXCR7, CXCL12) is known to play a pivotal role in the process of cell adhesion and metastasis [49]. Hyaluronic-acid receptor CD44 does not only participate in extracellular matrix interaction and migration, but is also a stem cell marker and highly involved in vasculogenic mimicry in ES [50]. CAV1 is a multifunctional membrane protein associated with malignancy in several tumors including ES [51,52] and regulation of pro-metastatic metalloproteinase MMP9 [53]. Moreover, CAV1 has been identified as gravity-sensitive protein in microgravity studies with endothelial cells [54,55] and thyroid cancer cells [24]. It is the aim of this study to identify the role of these genes and proteins, together with their associated regulators, in cell adherence under s-µg. A better understanding of the processes involved in self-aggregation of certain tumor cells to a three-dimensional spheroid, while other cells remain attached under the same circumstances is certainly beneficial not only to microgravity researchers but cancer researchers as well and could expand our horizon on characteristics of tumor cell adherence and their behavior in in vitro three-dimensionality [56] and could potentially contribute to new therapeutic approaches or targets.

## 2. Results

### 2.1. Light Microscopy

During the course of the observation period, a considerable fraction of A673 cells demonstrated detachment from the regular culture monolayer and formation of spheroids after 24 h. Mainly, these spheroids were uniformly sized, with some outliers to the smaller end consisting of less than 10 cells and a few outliers to the upper end exceeding the usual range by a several magnitudes. They exhibited mostly round to oval shapes even though more bizarre formations were observed as well. We commonly saw purportedly individual cells being attached to the outer perimeter of the spheroids like small round appendages. Each spheroid itself was composed of small round-shaped cells (Supplementary Figure S1). The 1 g control group grown under standard cell culture conditions showed typical flat, polygonal morphology with spiky cell processes. Adherent cells, which were exposed to s-µg but remain attached to the culture flask surface, seemed to be of an intermediate phenotype. Their growth pattern was still flat but they presented themselves much more round-shaped with fewer cellular processes, their density appeared diminished and less confluent with a tendency to grow more separately compared to the 1 g control group even though the amount of cells in each flask at the start of the 24 h experiment was equal. Neither spheroids, nor adherent cells showed light microscopic signs of impaired viability.

### 2.2. Trypan Blue Staining

**Figure 1:**
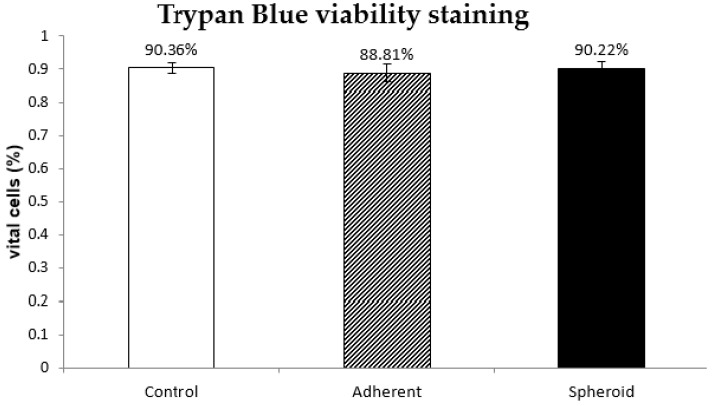
The diagram shows the percentage of vital cells quantified via trypan blue staining with a regular hemocytometer in each of the three groups (control, adherent, and spheroid) after 24 h of simulated microgravity (*n* = 5).

Trypan blue staining at 24 h revealed no difference in cell viability between 1 g control group, adherent cells under s-μg and spheroids. (*n* = 5) (Figure 1).

### 2.3. Confocal Microscopy after Actin Staining

**Figure 2:**
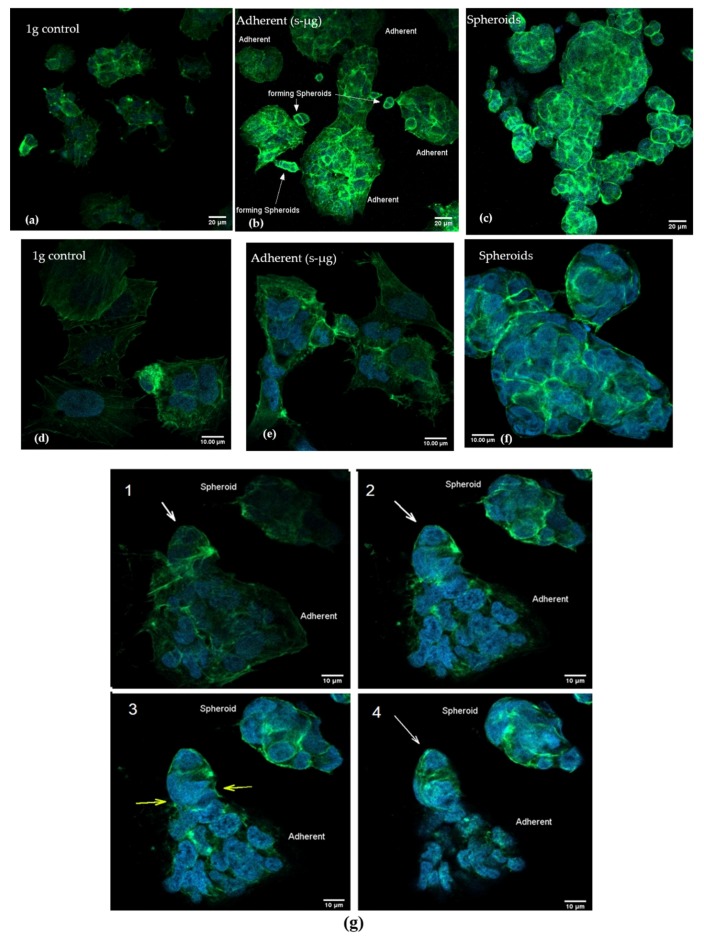
This figure shows 1 g control, adherent cells and spheroids with lower (**a**–**c**) and higher magnification (**d**–**f**). (**a**,**d**) The 1 g control group shows an acuter and spikier cell shape and a more longitudinal alignment of actin filaments. Under higher magnification (**d**), the longitudinal alignment becomes even more apparent. (**b**,**e**) Under simulated microgravity, cells exhibited less spikes and a generally rounder shape with diminishing longitudinal fibers (adherent cells) or, (**c**,**f**) in spheroids, almost no longitudinal fibers at all. After 24 h of s-µg, spheroids already form three-dimensional structures with peripheral actin arrangement around the area of the cell membrane. (**f**) Especially under higher magnification, the oval to round cell shape and a seemingly increased nuclear-cytoplasmic ratio becomes apparent. (**g**) shows the formation of a new, small spheroid (white arrow) which develops out of an adherent cell after 24 h of s-µg. Pictures show slices of cells from bottom to top (1–4). Yellow arrows mark the area of the adherent cell where separation occurs. The change in cytoskeleton from longitudinal actin fibers (1) toward more spherical and peripherally located actin filaments (2–4) becomes evident as the sectional plane moves upward.

After 24 h, A673 cells displayed rearrangement of actin filaments in a spherical fashion with accumulation of actin fibers in the periphery of the nucleus, which was most pronounced in the area of the cell membrane. The shape of the spheroids was oval to round with little to no cellular processes. Almost no linear actin filaments could be spotted in spheroids. Even though other signs of cytoskeleton rearrangement like discrete membrane blebbing or intracytoplasmic holes could be observed, these alterations were not considerably different from the control group or adherent cells under s-µg. It was noteworthy that spheroids seemed to have higher nuclear-cytoplasmic ratios than non-detached cells. (Figure 2c,f)

A673 cells under regular 1 g conditions grew flatter and exhibited more cell processes giving them a polygonal, spikier appearance. They displayed longitudinal alignment of actin filaments throughout the entire cytoplasm. Three-dimensional optical sectioning through the use of pinholes revealed that the most prominent longitudinal actin filaments could be found on the lower pole of the cells where they were attached to the underlying culture flask (Figure 2a,d).

Adherent cells grown under s-µg conditions represented an intermediate phenotype showing considerable longitudinal actin filaments, especially in their lower pole. For the most part their aspect seemed more obtuse or already round. In the intracytoplasmic department, a significant amount of intracellular holes and stress fibers could be found (Figure 2b,e).

### 2.4. Real-Time PCR

**Figure 3:**
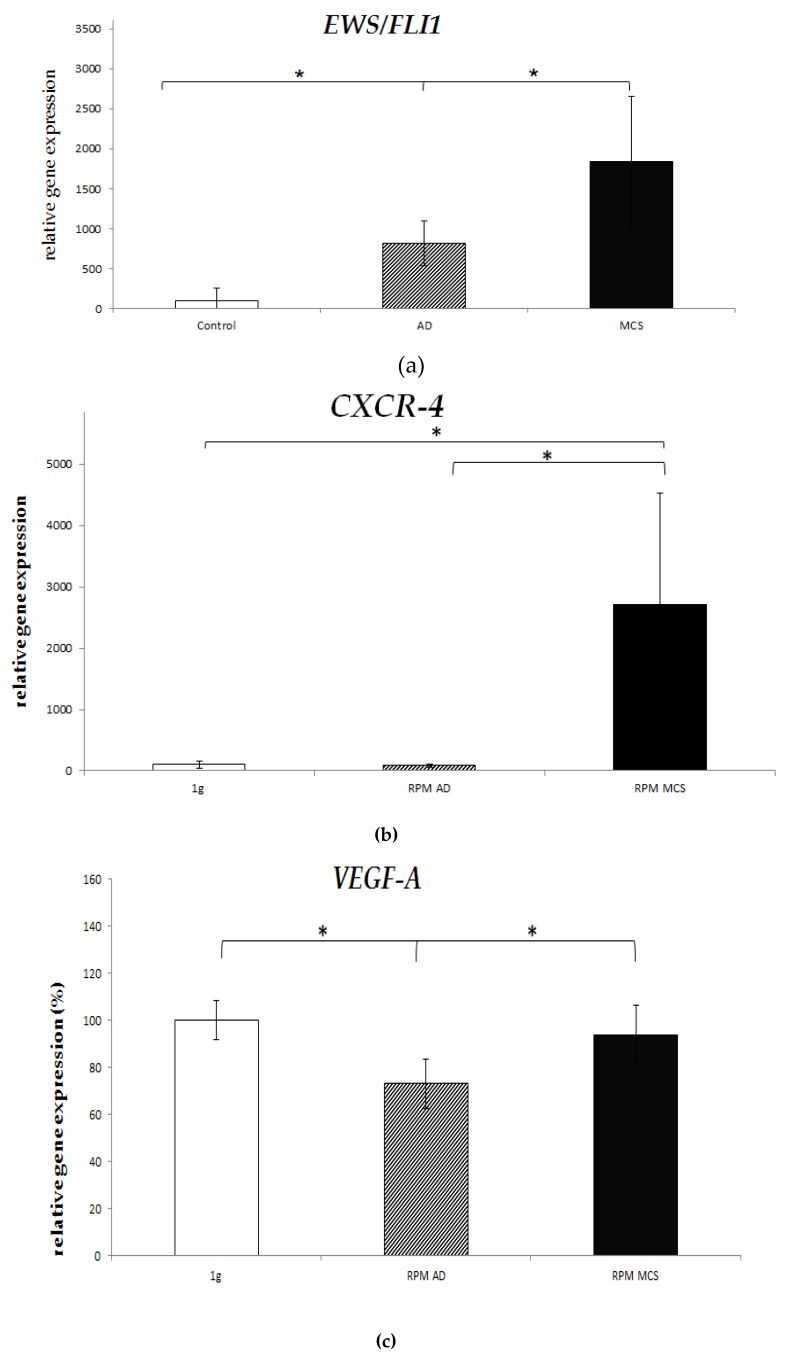

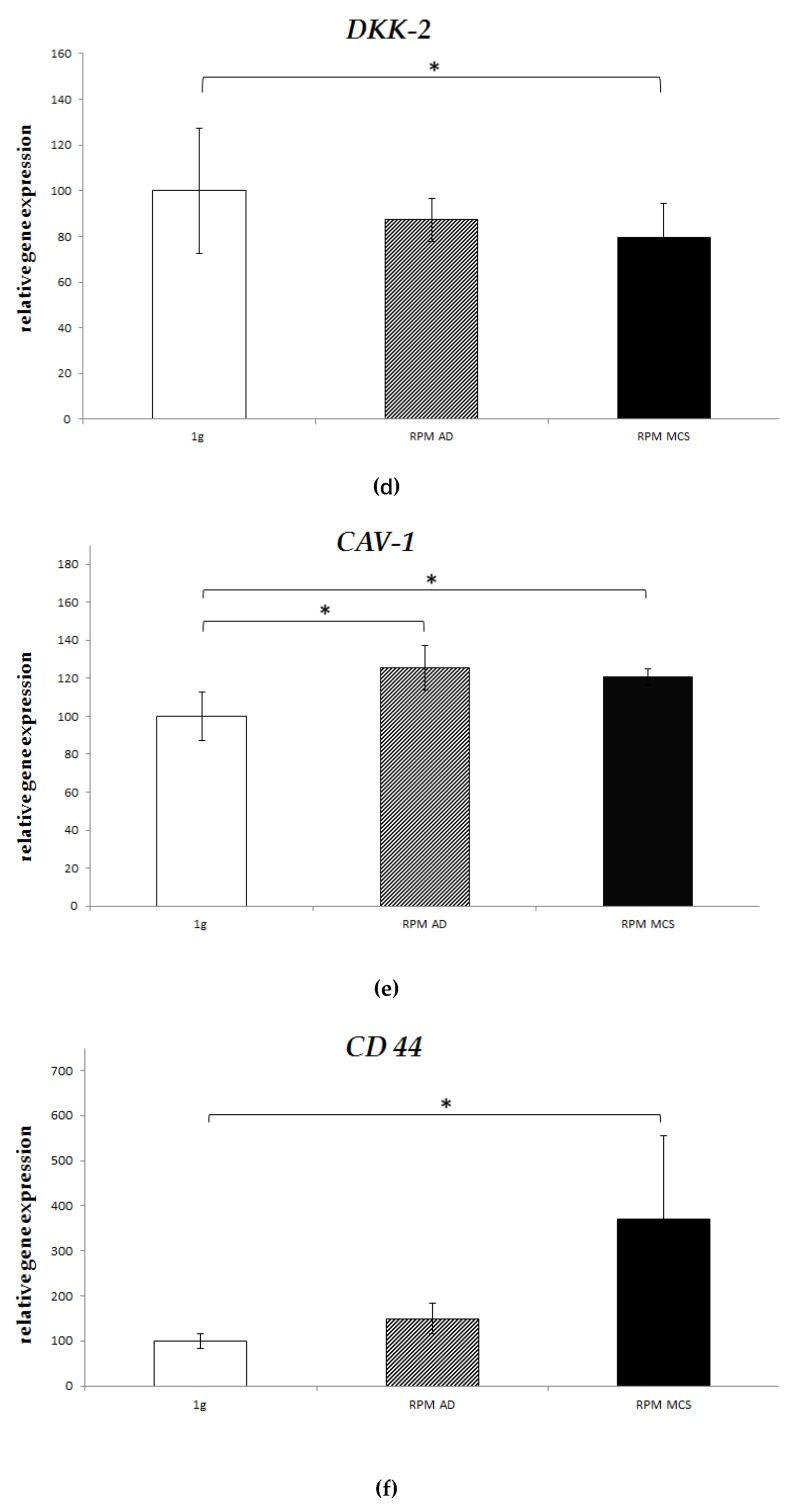
(**a**) *EWS/FLI1* expression was significantly upregulated in both spheroids and adherent cells compared to the 1 g control group (18.5x, 8.2x, *p* < 0.05 each). (**b**) *CXCR4* was significantly upregulated in spheroids compared to control and adherent cells (27x, 30x, * *p* < 0.05 each). (**c**) *DKK2* was downregulated in spheroids (0.8x, *p* < 0.05). (**d**) *VEGF-A* expression significantly decreased in adherent cells under s-µg (0.73x, * *p* < 0.05). (**e**) *CD44* was significantly upregulated in spheroids after 24 h (3.7x, * *p* < 0.05). (**f**) *CAV1* was significantly upregulated in spheroids and in adherent cells (1.3x, 1.2x, each * *p* < 0.05).

After 24 h under s-µg the tumor-specific main translocation-protein *EWS/FLI1* was significantly upregulated in both spheroids and adherent cells compared to the 1 g control group (18.5x, 8.2x, *p* < 0.05 each) (Figure 3a; Table 1). This was also significant when comparing spheroids with the adherent, non-detached cells. Whereas expression of *ZEB2* is not altered at all. The chemokine receptor *CXCR4* was significantly upregulated only in spheroids compared to control and even adherent cells (27x, 30x, *p* < 0.05 each.) (Figure 3b; Table 1). However, *CXCR7* expression was unaffected (Table 1). *DKK2* was downregulated under s-µg conditions but significance was reached only for spheroids (0.8x; *p* < 0.05) (Figure 3c, Table 1). *VEGF-A* expression remained constant in the control group as well as in spheroids, but significantly decreased in adherent cells under s-µg (0.7x; *p* < 0.05) (Figure 3d, Table 1). Hyaluronic acid receptor *CD44* was significantly upregulated in spheroids after 24 h (3.7x; *p* < 0.05). Adherent cells only showed an insignificant 1.5x increase in gene expression. (Figure 3e; Table 1) *CAV1* was significantly upregulated in spheroids as well as adherent cells (1.3x, 1.2x, each *p* < 0.05) (Figure 3f; Table 1). Additionally, 24 h of s-µg lead to a insignificantly decreased gene expression in spheroids and adherent cells for *IGFR1* (both 0.8x; Supplementary Figure S2a; Table 1), *LOX* (both 0.6x; Supplementary Figure S2c; Table 1), *ERBB4* (0.8x, 0.9x; Supplementary Figure S2f; Table 1) and *MMP9* (0.9x, 1.0x; Supplementary Figure S2b; Table 1). No changes in gene expressions occurred in *CD99* (Supplementary Figure S2h; Table 1). *NKX2.2* expression showed diverging results with slight upregulation in spheroids (1.2x) and downregulation in adherent cells (0.9x) but both failed to reach statistical relevance (Supplementary Figure S2d; Table 1). Expression levels of *E-Cadherin* were almost not detectable.

### 2.5. Western-Blot

**Figure 4:**
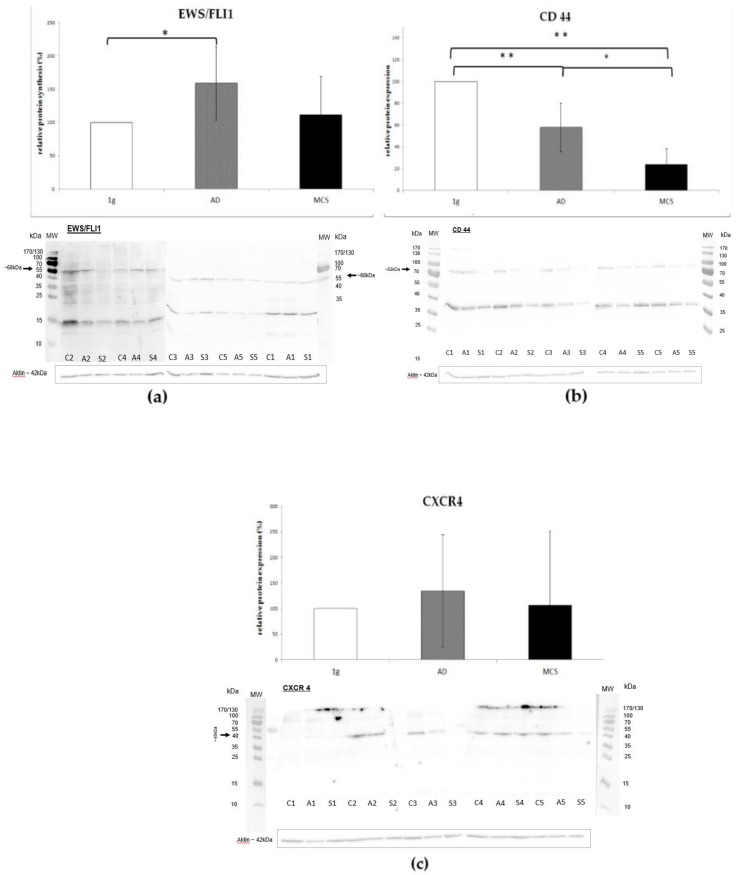
(**a**) Protein accumulation of EWS/FLI1 was significantly increased in adherent cells under s-µg compared to the control group (1.6x, * *p* < 0.05), the bar graph shows the average density of the blots from the respective experimental group (controls, adherent cells under simulated microgravity, spheroids) with the control group defined as 100%. Displayed below there are the corresponding western blot bands (molecular weight 68 kD). The protein of a corresponding independent experiment (1–5) is blotted next to each other as a triplet containing the corresponding 1 g control (C), adherent cells under s-µg (A), and spheroids (S). (**b**) In spheroids and adherent cells s-µg lead to a highly significant decrease in the protein accumulation of the standard CD44s-isoform at approximately 82 kDa (0.2x, 0.6x, ** *p* < 0.01) which was also significant comparing both groups directly with each other (* *p* < 0.05). The bar graph shows the average density of the blots from the respective experimental group (controls, adherent cells under simulated microgravity, spheroids): Below the corresponding western blot bands are shown (molecular weight 82 kD). The lighter band at approximately 37kDa corresponds to the unglycosylated CD44 core-protein. The protein of a corresponding independent experiment (1–5) is blotted next to each other as a triplet containing the corresponding 1 g control (C), adherent cells under s-µg (A), and spheroids (S). (**c**) CXCR4 protein accumulation was not significantly altered after 24 h of s-µg, the bar graph shows the average density of the blots from the respective experimental group (controls, adherent cells under simulated microgravity, spheroids) with the control group defined as 100%. Below the corresponding western blot bands were located at a molecular weight of 45 kD. The protein of a corresponding independent experiment (1–5) is blotted next to each other as a triplet containing the corresponding 1 g control (C), adherent cells under s-µg (A) and spheroids (S).

The relative protein accumulation of EWS/FLI1 was significantly increased in adherent cells under s-µg compared to the control group (1.6x, *p* < 0.05). Protein accumulation of EWS/FLI1 was also only slightly higher in the spheroids (1.1x) yet not statistically significant (Figure 4a). In both experimental groups (spheroids and adherent cells), there was a highly significant decrease in the protein accumulation of standard CD44s-isoform (0.2x, 0.6x, *p* < 0.01) (Figure 4b). CXCR4 protein accumulation was not significantly altered after 24 h of s-µg (Figure 4c).

### 2.6. Morphologic and Quantitative Analysis of CXCR4-Inhibition with Plerixafor (AMD 3100) after 24 h RPM-Exposure

**Figure 5:**
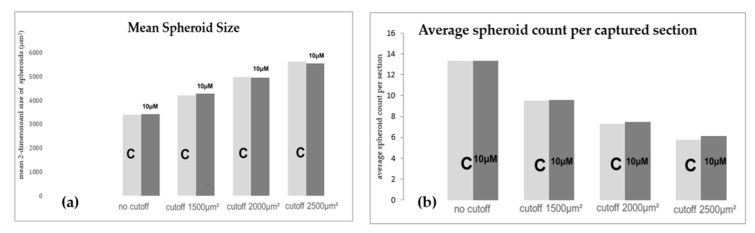
(**a**) Mean spheroid size was similar in 10 μM Plerixafor group and control group, even after applying different cutoffs after 24 h in s-μg. (**b**) The average count of spheroids per captured section did not differ between both groups.

After 24 h both groups, the 10 µM plerixafor experimental group and the control group, showed no visible differences regarding growth pattern, viability, size, or amount of spheroids. Counting and measuring each spheroid in numerous sections of each flask, the average number of spheroids was 13 spheroids/per section for both, the plerixafor and the control group. The mean area as a two-dimensional surrogate for the actual size of each spheroid was similar with 3406 μm^2^ for the control group and 3425 μm^2^ for the 10 μM Plerixafor group having counted and measured 845 and 825 spheroids, respectively. After applying cutoffs at 1500 μm^2^, 2000 μm^2^, and 2500 μm^2^ to sort out smaller spheroids in order to focus on bigger, more relevant spheroids, there was still no evident difference in mean size or spheroid count. With a cutoff at 1500 μm^2^, mean size was 4219 μm^2^ for control group and 4279 μm^2^ for the Plerixafor group, and the spheroid count was 615 vs. 603 spheroids. Application of a cutoff at 2000 μm^2^ led to 478 spheroids with an average size of 4982 μm^2^ vs. 475 spheroids with a size of 4953 μm^2^. Finally, we a looked only at spheroids bigger than 2500 μm^2^ and we observed 383 spheroids averaging 5615 μm^2^ in the control group vs 390 spheroids averaging 5558 μm^2^. Figure 5 illustrates the comparison described above.

### 2.7. Protein-to-Protein Interaction Network via STRING Analysis

**Figure 6:**
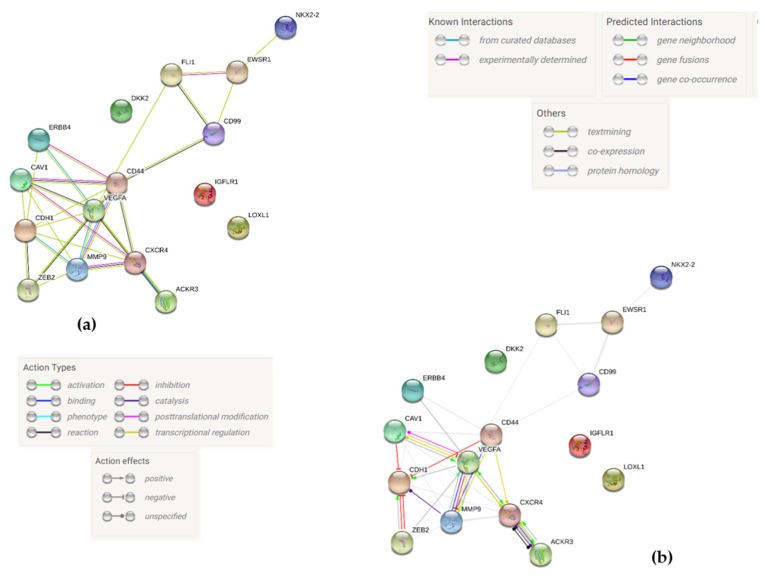
(**a**) String Map showing interactions between the different proteins whose expressions and/or protein concentration was measured after simulation of microgravity. Legend is added in picture. This Map was created by using STRING v11 [57], which was thankfully made freely available under a “Creative Commons BY 4.0” license. (**b**) String Map showing molecular pathways between the different proteins whose expressions and/or protein concentration was measured after simulation of microgravity. Legend is added in picture. This Map was created by using STRING v11 [57], which was thankfully made freely available under a “Creative Commons BY 4.0” license.

To analyze the interactions between the corresponding proteins of the genes tested we used the bio-informatic STRING (search tool for the retrieval of interacting genes/proteins) network and database, which processes, scores and integrates all publicly available sources of information for protein-protein interactions [57]. The following gene were integrated into the STRING Network with their UniProtKB identifiers: EWSR1 Q01844), FLI1 (Q01543), CAV1 (Q03135), CXCR4 (P61073), DKK2 (Q9UBU2), VEGFA (P15692), CD44 (P16070), IGFLR1 (Q9H665), MMP9 (P14780), LOXL1 (Q08397), NKX2.2 (O95096), ZEB2 (O60315), ERBB4 (Q15303), ACKR3 (P25106), CD99 (P14209), CDH1 (P12830). The STRING analysis (Figure 6) shows a network consisting of 16 nodes with 30 edges and a protein-protein-interaction enrichment value of 8.0 × 10^−5^, which means that there are significantly more interactions than expected. There is a group of eight proteins (VEGFA, CD44, CXCR4, MMP9, CDH1, CAV1, ACKR3, ZEB2), that’s profoundly interconnected with each other, while three gene products (DKK2, IGFLR1, and LOXL1_ are completely disconnected from each other and the rest of the proteins. The most central node is VEGFA. It affects CD44, CXCR4, and CDH1 positively but has inhibitory effects on MMP9. Furthermore, it has transcriptional effects on CXCR4, ACKR3, CAV1, and MMP9 and is involved in post-transcriptional modification of CAV1. CD44 also shows several interactions to other nodes: It is involved in transcriptional regulation of CXCR4 and MMP9, is able to bind MMP9 and inhibits CDH1. CXCR4 is object to transcriptional regulation by CD44 and VEGFA but also by chemokine receptor AKCR3, with which it interacts strongly with catalysis and reaction. Notably, CDH1 is the most inhibited node in the interaction network. It is inhibited by CAV1, CD44, and ZEB2.

## 3. Discussion

In our experiments, we observed several interesting results in ES cells under s-µg. As important findings, tumor marker EWS/FLI1 was more intensely expressed on the gene and protein level, and CXCR4, although highly upregulated under s-µg, does not seem to affect spheroid formation, as we showed by its inhibition.

Initially, we observed that s-µg reliably induces spheroid formation in human ES cells. Further, microgravity conditions lead to various significant changes in gene expression and amount of protein of factors involved in cell adhesion, angiogenesis, survival and proliferation. After 24 h, we observed detachment of the cells from their adherence to the culture flasks and formation of three-dimensional spheroid structures. This phenomenon is known from several other cell types before, such as tenocytes [20], lung cancer cells [58], thyroid cancer cells [24], and several others. This is indicative for a profound change in cell adhesion behavior, going from attachment to a surface, over release, toward three-dimensional intercellular adherence. Correspondingly, we observed a profound alteration of actin fiber alignment during the process of cell detachment and re-adhesion in spheroidal shape. During 1g conditions, cells show a longitudinal actin fiber alignment, changing into a more spherical shape, accentuated in the area of the cell membrane under s-µg. As it is known, the actin skeleton not only maintains cell stability, but also mediates a variety of cell-matrix and cell-cell-interactions. At the cell membrane, actin filaments are linked to E-cadherines, which are integral parts in adherence junctions and therefore mediate intercellular adhesions. [59,60]. Some reports have speculated that the actin skeleton could act as a kind of “gravity sensor“ that transduces external forces into the inner cell [61,62]. Furthermore, the cytoskeleton is known to react sensitively to external mechanical forces, partly mediated by Rho-family GTPases, partly mediated by mechanisms still unknown [63]. Tendon cells, e.g., have shown longitudinal alignment of their actin fibers under fluid shear forces imposed on the medium in a cell bioreactor [64]. By applying s-µg, we achieved an opposite effect, with a transformation from a longitudinal to a spherical actin orientation. With the existing knowledge about actin as a part of the intercellular adhesion system, this change in actin alignment under s-µg seems likely to affect cell adhesion during spheroid formation. The exact mechanisms, however, still need to be elucidated.

In addition to the alterations in cytoskeleton shape, we observed various changes in gene expression and protein accumulation. EWS/FLI1 is an oncogenic fusion protein and transcription factor present in about 85% of all ES tumors [65]. Sustained expression of EWS/FLI1 is necessary to maintain the phenotype of this tumor, and EWS/FLI1 inhibition has been shown to reduce oncogenic transformation [66]. EWS/FLI1 knockdown by RNA interference has been shown to have tremendous effects on Ewing´s sarcoma cell motility and adhesion [47]. In a 2D environment, increased EWS/FLI1 expression has been shown to reduce cell adhesion [47]. This can be seen in accordance with our results, where detachment of the cells from the culture plate is associated with an increased EWS/FLI1 expression. The spheroidal state is obviously characterized by a different type of adhesion that occurs even under high expression levels of EWS/FLI1. In our experiments, we observed significantly upregulated gene expression and significantly increased protein accumulation of EWS/FLI1 at least in the adherent cells under s-µg. This might indicate an improved phenotype preservation of Ewing´s sarcoma under these conditions. As it is known, several tumor cell types show better phenotype preservation under 3D compared to 2D culture conditions [67,68]. For ES in particular, 3D culture models have shown to mimic in vivo conditions better than conventional 2D culture [69]. Furthermore, ES cells have been shown to react sensitively to mechanical forces, particularly regarding gene expression. Shear stress induced by medium flow has been shown to induce insulin-like growth factor 1 (IGF-1) ligand production, while HER2 production was decreased [70]. Accordingly, by reduced external forces under s-µg, we observed a variety of alterations in gene expression. CD44 has been reported to mediate enhanced adhesion of Ewing´s sarcoma cells to the extracellular matrix ligand hyaluronic acid [50,71]. Overexpression of CD44 is supposed to be related to higher aggressiveness of the tumor. In our experiments, we found an upregulation of CD44 on mRNA, but not on protein level. The exact reasons for this are to be further elucidated. A lacking positive feedback mechanism from the original ligand hyaluronic acid in the ECM-free environment, or delayed protein synthesis at a later time point could be conceived, however. DKK2 is a gene that is highly overexpressed in human ES cells. It is supposed to be a key player in invasiveness and metastatic potential of the tumor. It is further known to regulate expression of CXCR4 among others [72]. We found some minor decrease in DKK2 gene expression in the spheroids (0.8x). This difference was calculated to be statistically significant but seemed of minor practical importance to us so that we refrained from western blot confirmation. A lack of upregulation of this gene under s-µg might hint at the existence of some alternative mechanisms regulating adhesion of ES cells under s-µg. Further experiments have to identify these mechanisms in more detail, also with regard to a potential role in metastasis formation in vivo. CXC-chemokine receptor-4 (CXCR4) is known to play a role in cell adherence and metastasis formation of various tumors [73]. It binds to its ligand CXCL-12, what triggers several signal transduction pathways. Particularly, CXCR4 activation is related to cell migration and adhesion. Especially in ES, CXCR4 is known to be highly sensitive to micro-environmental factors like hypoxia [74], serum deprivation, confluent growth conditions [75], and concentration of CXCL12 [76]. It is believed that CXCR4-mediated metastasis formation happens in a three-step process involving autocrine, paracrine and endocrine loops. First, ES cells are induced to upregulate CXCR4 in an paracrine or autocrine fashion. Second, CXCR4-overexpressing cancer cells can then migrate along CXCL12 gradients. Third, overly high CXCL12 levels lead to receptor internalization and subsequent stay at the site of metastasis. [76,77]. Intriguingly, CXCR4 also plays a critical role in spheroid formation in sphere formation essays used for isolation of cancer stem cell subpopulations, where CXCR4-inhibition drastically reduces sphere size and sphere count in some tumors [78,79]. We therefore measured CXCR4 expression both on mRNA and on protein level. Indeed, we found a strong upregulation of CXCR4 mRNA expression in the spheroids, with a slight increase in CXCR4 protein levels in the adherent cells under s-μg but without statistical significance. For the discrepancies we observed between mRNA expression and protein levels of CXCR4, but also EWS/FLI1 and CD 44, several reasons can be conceived. On the one hand, a general tendency of a decreased protein synthesis under microgravity has been described before [80,81,82] for reasons yet to be evaluated. On the other hand, a propensity towards increased protein degradation under µg has been reported in literature [83,84,85]. Independent from that, several post-trancriptional mRNA modifications exist that influence further translation. Among those modifications, small non-coding miRNA [86,87] and RNA-binding proteins (RBPs) [88] are probably the most notable and take a profound and intricate role mainly in inhibition of protein synthesis, controlling and influencing about 30% of coding genes [89]. As a matter of fact, an increasing number of studies shows that microgravity, indeed, alters miRNAs considerably [90,91,92]. Another possible explanation could be different half-lives of proteins under s-µg [93] and delayed protein synthesis or rapid degradation of mRNA, e.g., via miRNAs as it is discussed above. Furthermore, it is possible that the set in of microgravity, which is a known stressor to mammalian cells, caused a stress reaction in tumor cells, protein synthesis stalled and has not yet caught up with an already reconstituting gene expression. If this was true, further experiments with longer time frames than 24 h would be necessary to show a delayed convergence of gene expression and corresponding protein level data. In the same vein goes the theory of an unfolded protein response (UPR) to microgravity, which induces stress resulting in an initial global inhibition of protein synthesis. This mechanism has been described for cardiac myocytes of rats under s-µg, e.g., reference [80], would counteract the strong upregulation in gene expression and becomes evident in the highly significant downregulation of CD44 and relatively unchanged protein levels of the highly overexpressed genes EWS/FLI1 and CXCR4. Taking these factors into consideration, it becomes apparent that many processes happening under microgravity are not fully understood yet or can merely be abstracted for ES, as no one has ever cultivated ES cells under simulated microgravity before. Hence, further experiments are necessary in order to fully explain our findings and create a more coherent picture of ES behavior in microgravity.

Taken the existing knowledge of CXCR4 as a key player in tumor cell adhesion and sphere formation, we found it worth to examine the effect of its inhibition on spheroid formation under s-µg, particularly as its gene expression was significantly upregulated in the spheroids. We utilized CXCR4 antagonization by plerixafor (AMD3100) under the hypothesis that it might decrease spheroid formation in ES cells as well. Plerixafor as a CXCR4 inhibitor has been in clinical use in anticancer therapy for over ten years now, with approval for Non-Hodgin´s lymphoma, but also with promising experimental results for several other solid tumors such as thyroid cancer, pleural mesothelioma, or ovarian cancer [94]. In an animal model of cervical cancer, CXCR4 inhibition by plerixafor lead to better sensitivity to radiotherapy and reduced metastasis formation [95]. With regards to spheroid formation, CXCR4 seems to play a particularly important role as CXCR4 inhibition, e.g., with plerixafor (AMD3100), profoundly reduces size and number of 3D spheres in sphere formation essays [78,79]. Surprisingly, despite its considerable, significant upregulation at mRNA level and light increase in protein accumulation in the adherent cells, CXCR4 inhibition did not alter neither spheroid size nor their number after 24 h of s-µg. Although the exact mechanisms are still unclear several reasons for this finding can be thinkable. As mentioned above, several post-translational modifications can be conceived that alter CXCR4 function, as well as post-transcriptional modifications, that may explain the discrepancy between mRNA expression and protein levels. Furthermore, cell adhesion under microgravity is a highly complex, involving several pathways such as mTORC or ERK1/2 [96]. Further, still unknown mechanisms that mediate spheroidal adhesion under simulated microgravity in Ewing’s sarcoma, independent from CXCR4, can therefore be postulated.

We are aware that our experiments were conducted only under a simulation of microgravity and that our findings cannot simply be applied to real microgravity. However, as real microgravity for a duration of 24 h can only be achieved in space, where access is limited and expensive, there is a need for alternatives to do gravity research or pilot experiments before ultimately conducting them in space. As we performed our studies on the RPM, a ground-based facility, gravity is naturally effective on our samples. Yet, by constantly changing the direction of the gravity vector relative to the samples by means of two gimbal-mounted axes and dedicated algorithms, we achieved a convergence of the gravity vector to zero over time [97]. For several cells and organisms similar effects of real microgravity and simulated microgravity via RPM have already been described [98,99,100,101], so we are confident to have utilized a suitable simulation model.

Finding a “key effector” that mediates the mechanisms of spheroidal cell adhesion under s-µg could deliver valuable information about tumor behavior in weightlessness and their invasiveness. This knowledge might reveal hitherto unknown information about cell aggregation and cluster formation and could potentially contribute to new therapeutic approaches or targets.

A number of further genes that we tested with qRT-PCR showed no changes under s-µg. These were LOX, MMP9, ZEB2, NKX.2.2, ERBB4, E-CADHERIN, CD99, CXCR7, and IGF1-R. We tested these genes, as many of them are related to adhesion and metastatic behavior (LOX [102], MMP9 [103], ZEB2 [104], ERBB4 [105], E-CADHERIN [106], CXCR7 [107]), and others are related to phenotype expression (NKX2.2 [108], CD99 [109], IGF1-R [110]). Particularly, NKX2.2 and CAV-1 are known to be target genes upregulated by EWS/FlI1 [52,111]. In our experiments, we did not observe such an upregulation in *NKX2.2*, and an upregulation of *CAV-1* that was calculated as significant, but too low to have practical importance, despite increased expression of EWS-FLI1 under s-µg. This could be explained by our observation time point, when a delayed upregulation is postulated, or by alternative mechanisms of gene regulation under s-µg that are yet to be explained.

By measuring gene expression of ZEB2, we addressed an explanatory approach for Ewing’s sarcoma metastasis formation via cellular plasticity done by Wiles et al. [48]. These authors speculate that the ability of ES cells to strongly proliferate at the primary site while still having the tendency to metastasize early and extensively lies in a functional antagonism between EWS/FLI1 and ZEB2. According to this theory, EWS/FLI1 inhibits mesenchymal differentiation while ZEB2 simultaneously inhibits epithelial differentiation. As ZEB2 expression remains unaffected after 24 h of s-µg, we conclude that the functional antagonism between EWS/FLI1 and ZEB2 probably does not play an important role in short-term spheroid formation under s-µg.

A semantic analysis of corresponding protein-protein interactions of the genes which have been investigated by our group identifies CD44 and VEGF-A as the main hub of interactions and CXCR4, CAV1, and MMP9 as important players. Notably, these proteins not only seem to occupy central roles but are shown to all have intertwined interactions with each other. It is interesting, that the two individual genes EWSR1 and FLI1, which fuse together to build the pathognomonic EWS/FLI1 oncogene in ES, only have marginal roles on their own, contrastingly to the immense effect their fusion protein exerts on gene up- and downregulation.

Further, the central role of VEGF-A becomes apparent when focusing on the activating or inhibiting relationships between each protein in this STRING interaction network (Figure 6b), as VEGF-A largely activates proteins located around it in the STRING map, except for MMP9 which is inhibited by VEGF-A. Notably after 24 h under s-µg, our group found a significant decrease in *VEGF-A* expression only in cells which did not detach to build spheroids. Given its pivotal position, the general role in tumor angiogenesis and growth and its reproducible responsiveness towards r- and s-µg in different cell types [26,45,112], evaluation of VEGF-associated pathways might be worthwhile.

CXCR4 and CXCR7 (ACKR3) are strongly interconnected with each other. Yet, despite reciprocal upregulation (reciprocal green arrow in Figure 6b) between the two, s-µg only significantly increases *CXCR4* expression whereas *CXCR7* expression remains unaffected. A yet unknown key mechanism in spheroidal cell adhesion and detachment, that influences CXCR4 without influencing CXCR7 might be insinuated, as it already mentioned above.

Fittingly, CDH1, which is almost not detectable with qPCR in both the experimental and the control groups, is the most inhibited node in the STRING map as well.

Another strong interaction can be found in hyaluronic-acid receptor CD44, localized at the cell surface, and matrix metalloproteinase MMP9. It has been shown that both are not only highly involved in invasiveness and migration on their own [53,113,114,115] but also collaborate. CD44 provides a surface docking station for active proteolytic MMP9; this specific localization at the cell membrane is required for MMP9’s ability to promote tumor invasion. [116]. Consequently, the STRING map displays an activating impact of CD44 on MMP9, which has been shown to inversely activate CD44 by cleavage into a more active form as well and thus promoting cellular motility [117]. Interestingly, s-µg affects both players differently. Under 24 h of s-µg, MMP-9 expression showed no significant alterations while CD44 gene expression as well as protein accumulation were significantly influenced. Yet, it is conceivable that the co-localized CD44/MMP9-complex still confers an altered invasive or migratory capacity under s-µg despite the fact that MMP9 is unaffected by s-µg. In future experiments, it might be worthy to intensify the focus on this functional gene pair in order to characterize their interplay in s-µg, test whether aforementioned cell-membrane-located complexes still exist when gravity and – as we showed in our experiments –parts of the cytoskeleton are altered, and to illuminate their overall effect on migratory capacity in s-µg compared to 1g.

## 4. Materials and Methods

### 4.1. Cell Culture

Human Ewing’s sarcoma cancer cells (ATCC^®^ CRL-1598) were obtained from ATCC© (Wesel, Germany). Cells were first expanded under regular cell culture conditions in T75 flasks (Sarstedt, Nümbrecht, Germany). Ham’s F12-media (Gibco, Berlin, Germany) supplemented with 10% fetal calf serum (FCS) (Biochrom AG, Berlin, Germany) and 1% penicillin/streptomycin (Biochrom AG, Berlin, Germany) was used. Incubation took place in 95% air and 5% carbon dioxide at 37 °C. Medium was changed three times per week and subcultivation was carried out in a 1:3 to 1:6 ratio with 0.25% trypsin 0.03% EDTA (Gibco, Berlin, Germany). For the qPCR experiments, 1.5 × 10^6^ cells were counted by hemocytometer and added to *n* = 6 T25 flasks for the experimental group in the random positioning machine and *n* = 5 T25 flasks for the control group under 1g conditions. For Westernblots, we aimed for 1.5 × 10^6^ cells in *n* = 5 T25 flasks in both the control and the RPM groups. For cytoskeleton staining, cells were seeded with a density of 1 × 10^5^ per cm^2^ to slide flasks (Thermo Scientific, Darmstadt, Germany). The Plerixafor inhibition experiments were performed with 1.0 and 1.5 × 10^6^ cells per T25 flask (*n* = 5). Vitality testing via trypan blue was performed under regular experimental conditions with 1–1.5 × 10^6^ (*n* = 5).

### 4.2. Random Positioning Machine

The RPM (developed by University of Applied Sciences, Northwestern Switzerland) was run with a commercially available incubator (Galaxy 14S, New Brunswick, NJ, USA) located in the center of two gimbal-mounted frames, which are moving independently from each other. This way, the samples inside the incubator are constantly reoriented in a random manner and the trajectory of the mean gravity force converges to zero (during 24 h experiments calculated gravity force was generally as low as 0.002 g). Specific algorithms assure that the rotation of frames is faster than the biological process studied, but not too fast to let centrifugal forces distort the effects [30]. In order to avoid shear forces, we took meticulous care to avoid air bubbles inside the culture flasks. Inside the incubator, cells were incubated in 37 °C and 5% CO_2_. The device was operated in a random walk, constant speed modus using an angular velocity of 60 °/s. The method and the RPM used with its algorithms and further specifications were intensively investigated and published earlier [32,97]. A maximum of T25 flasks were compactly attached to the operating platform positioned as centered as possible, and samples were rotated for the selected time period (24 h). Static, non-rotated controls were exposed to the same environmental conditions nearby the device. The RPM machine was checked multiple times a day and was rebooted once per 12 h to ensure proper operation. Interruption was kept as short as possible every time.

### 4.3. Phase Contrast Microscopy

Phase contrast microscopy was performed for visual observation of the viability and morphology of the cells, and for the detection of spheroids. A Leica microscope (Leica Microsystems GmbH, Wetzlar, Germany) was used. Pictures were taken with a Canon EOS 60D (Canon GmbH, Krefeld, Germany). Scaling was accomplished by correlation with known measurements of a generic hemocytometer.

### 4.4. Trypan Blue Vitality Staining

Trypan blue stock solution was obtained from Thermo Fisher (Berlin, Germany). Aliquots of the control and the experimental groups were taken at a volume 100 μL and mixed with an equal amount of trypan blue stock solution. The mixture was incubated for two minutes at room temperature and then loaded onto a hemocytometer in a volume of 100 μL. Cell count was performed and the relation of viable to non-viable cells was calculated.

### 4.5. Cytoskeleton Staining

Cells exposed for 24 h to simulated microgravity in the RPM in slideflasks were investigated. F-actin was visualized by means of phalloidin staining (PromoKine, High Point, NC, USA). Both adherent cells and spheroids were fixed with 4% paraformaldehyde for 10 min and permeabilized with 0.2% Triton-X (Sigma-Aldrich, St. Louis, MO, USA) for 5 min. After washing with PBS solution three times, nonspecific binding was blocked by incubation with 3% bovine serum albumine (BSA) for 1 h at room temperature. Staining was performed by incubation of the slides with 6.6 μM solution of a phalloidin/Alexa Fluor 488 conjugate for 60 min at room temperature, followed by thorough washing with PBS solution. Nuclei were counterstained with 4′,6-diamidine-2-phenylindol (DAPI (Thermo Fisher Scientific, Berlin, Germany) at a concentration of 0.1 μg/mL for 1 min. Afterward, the samples were mounted with Vectashield mounting medium (Vector Laboratories, Burlingame, CA, USA).

### 4.6. Confocal Microscopy

Confocal microscopy of the slides stained for F-actin was performed using a Zeiss 510 META inverted confocal laser scanning microscope (Zeiss, Oberkochen, Germany). Excitation and emission wavelengths for Alexa-Fluor and DAPI were 488nm/560nm, respectively. The three-dimensionality of spheroids and adherent cells in the experimental group as well as the control group was captured by scanning the entirety of the respective structures in pinhole technique, which created horizontal slices along the Z-Axis (depth of the slides) with slice thickness 0.28 μm in 630x magnification. Pinhole size was set at 1.15 AU. Laser intensity was kept as low as reasonably possible to allow for maximum discrimination of actin fibers.

### 4.7. RNA Isolation

Directly after harvesting the cells from the 24 h-RPM run cells were processed for subsequent RNA isolation using the RNeasy© Mini Kit (Qiagen, Hilden, Germany). Non-detached cells (1 g cells and monolayer cells under μg conditions) were carefully detached using a Cell Scraper 2-Position Blade 25 (Sarstedt, Newton, NC, USA). Spheroids were collected by pipetting the spheroid-containing cell medium into 50 mL conical tubes, in which they were then centrifuged and resuspended in Dulbecco’s Phosphate Buffered Saline (DPBS, Gibco, Berlin, Germany). The cell suspensions were transferred to RNAse-free 1.5 mL tubes (Eppendorf, Hamburg, Germany) and once again centrifuged for 5 min at 300 g. Subsequent lysis, homogenization and RNA-isolation were performed following the RNeasy© Mini Kit (Qiagen, Hilden, Germany) manufacturer’s manual. The pellet was lysed by adding 350 μL (1 volume) of the lysis buffer (RLT, Qiagen), which contained 1% β-mercaptoethanol for inactivation of cell-inherent RNases. Cell membranes were disrupted by vortexing for 1 min and homogenization was accomplished by thoroughly rinsing the lysate up and down through a sterile 1mL syringe with 25G cannula. It was then stabilized by adding 350 μL (1 volume) of 70% ethanol. Afterwards the solution was added to an RNeasy Spin Column and centrifuged in a microcentrifuge at 14,000 rpm for 15 s. The flow-through was discarded. Subsequently, 700 μL of the washing buffer RW1 was added to the spin column and the column was centrifuged again at 14,000 rpm for 15 s, and the flow-through was discarded. Then, 500 μL of the washing buffer RPE were added to the spin column and centrifuged at 14,000 rpm for 15 s. This step was repeated after discarding the flow-through to maximize the RNA-concentration. Finally, the spin column was placed into an RNase-free collection tube and the RNA was eluted with 30 μL of RNase-free water. The RNA was quantified photometrically with an Ultrospect 2100 Pro device (GE Healthcare, Boston, MA, USA). For that, 2 μL of RNA were used in a 1:50 dilution to determine the amount of isolated RNA by measuring the absorbance at 260 nm. Correlation with the absorbance at 230nm, 280nm and 320nm was done to rule out possible impurities. After determining RNA concentration, the RNA was immediately stored at −80 °C for further processing.

### 4.8. Reverse Transcription

Reverse transcription was performed using the First Strand cDNA Synthesis Kit (Thermo Scientific, Waltham, MA, USA) following the manufacturer’s instructions. For each sample, 0.5 μg total RNA and nuclease-free water were added to a total volume of 10 μL. To this mix, 1 μL random hexamer primers, 4 μL reaction buffer, 1 μL RNase inhibitor, 2 μL oligonucleotides, and 2 μL reverse transcriptase (M-Mulv) were added to a MicroAmp™ Optical 96-Well Reaction Plate (Applied Biosystems, Foster City, CA, USA) to a total volume of 20 μL. Afterward, the reaction plate was sealed with a MicroAmp™ Optical Adhesive Film (Applied Biosystems). All steps were carried out on ice. After mixing the solutions, the 96-Well Reaction Plates were inserted into a 7500 Fast Real-Time PCR System (Applied Biosystems). It incubated the reaction plate for 5 min at 25 °C, followed by the reverse transcription phase at 42 °C for 60 min. The reaction was terminated by incubation at 70 °C for 5 min. The cDNA solution was stored at −80 °C before proceeding with the experiments.

### 4.9. qRT-PCR

Quantitative real-time polymerase chain reaction was utilized to determine the expression levels of target genes, shown in Table 1, using the SYBR^®^ Green PCR Master Mix (Applied Biosystems, Darmstadt, Germany) and the 7500 Real-Time PCR System (Applied Biosystems, Foster City, CA, USA). Determination of expression was done in replicates of 3 for each probe and gene. A total of 14 μL master mix consisting of 7.5 μL of SYBR^®^ Green PCR Master Mix, 0.045 μL of forward and reverse primer stock solution each at a concentration of 100 μM, resulting in a final concentration of 300 nM, and 6.41 μL of RNA- and nuclease-free water was created. To every 14 μL mastermix containing reaction well of the MicroAmp™ Optical 96-Well Reaction Plate (Applied Biosystems) 1 μL of cDNA was added, the reaction plate was covered with MicroAmp™ Optical Adhesive Film (Applied Biosystems) and the PCR run was started. After a first holding stage at 50 °C for 2 min and s holding stage at 95 °C for 10 min, 40 replication cycles were performed: 15 s at 95 °C alternating with 1 min at 60 °C. To ensure specific amplification a melting curve was implemented for confirmation. In the process temperature was gradually increased by 1% up to 95 °C. The majority of cDNA-selective primers were identified in established publications by literature research and subsequently double-checked with US National Library of Medicine Primer Blast Software (https://www.ncbi.nlm.nih.gov/tools/primer-blast/) or in a few cases primarily acquired with the aforementioned software. All primers were synthesized by TIB Molbiol (Berlin, Germany). All samples were measured in triplicate and normalized to the housekeeping gene HPRT1, which was shown to be a reliable housekeeper for the A-673 Ewing’s sarcoma cell line [118]. The comparative CT (ΔΔCT) method was used for relative quantification of transcription levels, with the control group set as 100%. Primer sequences were as indicated in Table 2.

### 4.10. Western Blots

Gel electrophoresis, transblotting, and densitometry were performed according to standard protocols extensively described elsewhere [124]. Cell lysates were produced by thoroughly resuspending the frozen cell pellets with 100–200 µL 97 °C hot SDS sample stock buffer (6% 1M Tris-HCl at pH 6.8, 10% Glycerol, 2% SDS, 2.5% bromphenol blue mixed in ultrapure water) freshly added with 2.5% β-mercaptoethanol and 1X cOmplete™ EDTA-free protease inhibitor (Roche, Basel, Switzerland). Samples were denatured at 97 °C for 5 min and stored at −80 °C. Protein concentration was determined fluorescence-based using Protein Assay Kit Qubit with the Qubit fluorometer 2.0 (Life Technologies Corp., Carlsbad, CA, USA). Then, 30 µL probes each containing 40 µg of total protein (plus 60 or 100 µg for FLI1) were loaded together with the prestained page rule (#26616; Thermo Scientific, Waltham, MA, USA) onto a 10% SDS–polyacrylamide (8% SDS-polyacrylamide in case of CD44) gel, followed by electrophoresis and semi-dry blotting onto 0.45-μm nitrocellulose membranes (Whatman, Dassel, Germany). Primary antibodies (all obtained from Thermo Fisher) were used in blocking reagents (5%-milk-TBS-T for anti-CD44 and anti-CXCR4; 0,5%-milk-TBS-T for actin; 10mL of AdvanBlock-Chemi Blocking Solution (Advansta, Menlo Park, CA, USA) in concentrations as follows: mouse anti-CD44 (Catalog # MA5–13890; 1:133; 1.5 µg/mL) mouse anti-CXCR4 (Catalog # 35-8800; 1:250; 2 µg/mL) and mouse anti- FLI1 (Catalog # MA1-196;1:500; 2 µg/mL) and mouse anti-actin (A1978, Sigma-Aldrich, St. Louis, MO, USA; 1:500) as control. The sary antibody for anti-CXCR4 and anti-CD44 was purchased with the “BM Chemiluminescence Western Blotting Kit mouse/rabbit” (Roche, Basel, Switzerland) and used in a dilution of 1:500 in 0.5% of the manufacturer’s proprietary blocking solution. In case of FLI1 a HRP-coupled goat anti-mouse sary antibody (Biozym Scientific, Oldendorf, Germany) was used. Afterwards, the blots were stripped at 50 °C for 30 min with stripping buffer (Restore Western blot stripping buffer, Thermo Scientific, USA), washed and re-probed with anti-actin antibody (1:500, Cell Signaling, Frankfurt, Germany). Blots were detected and analyzed on an Alpha-Ease^®^ FC Imaging System (Alpha Innotech, Kasendorf, Germany). For blot analysis, bands were boxed-in with uniform rectangles following the instructions of the user manual. After inversion and auto-backgrounding, integrated density values were calculated using the SpotDenso Mode. Five independent experiments were performed, and each band represents results from one experiment.

### 4.11. Morphologic and Quantitative Analysis of CXCR4-Inhibition with Plerixafor (AMD 3100) after 24 h RPM-Exposure

**Figure 7:**
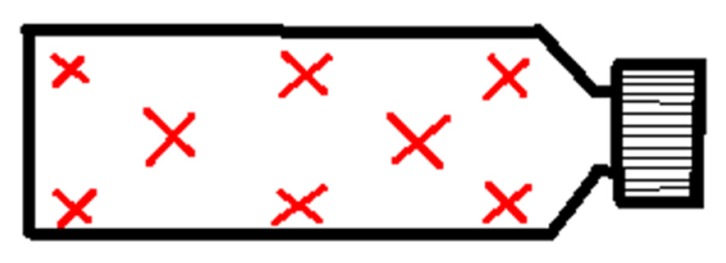
The red crosses correspond to the location of the first eight pictures taken in standardized fashion before another seven randomly located picture were taken for each cell culture flask.

To analyze macroscopic effects of CXCR4-inhibition on A673-Ewing sarcoma cells under µg conditions compared to 1 g conditions, the same 24 h-timeframe was chosen which was used before for qPCR-analysis and western blots. Cells were grown under accustomed cell culture conditions until the 24 h RPM experiment started. On the day of the experiment, the old cell culture medium was discarded, and T25-culture flasks were washed with PBS as usual. The CXCR-4 antagonist plerixafor (AMD 3100 octahydrochloride, Tocris Biosciences, Bristol, UK; Batch No. 2) was added to fresh regular cell culture medium (10% fetal bovine serum + 1% Penicilline/Streptomycin) for a final concentration of 10 µM, which has to be classified as a relatively high concentration based on several publications [78,79,125]. T25-flasks were filled bubble-free with the inhibitor-containing medium and the experiment started. After 24 h on the RPM culture flasks were turned upside down relative to the cell growth area to prevent attachment of spheroids to the adherent cell monolayer and thus create similar, undistorted conditions for analysis. Exactly 5 min before analysis started, each flask was turned onto the regular side to allow spheroids to sink down to the cell growth area in a uniform manner until pictures were taken. Plerixafor flasks and control flasks were processed alternately to rule out spheroids sinking time as confounder. Here, 15 total pictures in 10x magnification were taken of each culture flasks, the first eight in standardized fashion, as illustrated below in Figure 7, with the exact captured being random, and the other seven pictures were taken completely randomly.

Analysis was conducted using Fiji/ImageJ (Version 2.0). Before starting, a scale bar was implemented by using known measurements of a standard hemocytometer as reference. After setting the scale, each spheroid was meticulously encircled using the freehand selection tool and highlighted with a color to prevent spheroids from being accidentally counted twice. Afterward, the surface areas of the spheroids were measured as a two-dimensional surrogate of their actual three-dimensional size and data was subsequently transferred into excel (Microsoft, Redmont, Washington, USA) where further analysis was done. As spheroids are not defined strictly by size but by their nature of growing as a three-dimensionally organized structures detached of the regular monolayer, several cutoffs were implemented to potentially exclude structures, which are too small or are tinier spheroids with less of a physicochemical gradient.

### 4.12. Protein-to-Protein Interaction Network via STRING Analysis

The STRING protein interaction network is built to integrate all predicted and already established protein-protein association data to create a comprehensive global network of direct and indirect interactions. In part, it implements proven classification systems like Gene Ontology and KEGG. The network nodes represent proteins and connecting edges represent interaction between two proteins, that are meant to be meaningful and of importance. As it is illustrated in the legend attached to the STRING map, each color stands for different types of known or predicted interactions. Only functional interactions with at least a medium confidence score of > 0.400 were included in the networks. Final PPI-enrichment *p*-value was 8.0 × 10^−5^ meaning that the studied proteins have significantly more interactions than one would expect from randomly chosen proteins [57]. The map was created by using the latest version STRING v11 [110], which was thankfully made freely available under a “Creative Commons BY 4.0” license.

### 4.13. Statistical Evaluation

All statistical analyses were performed using SPSS 22.0 (SPSS, Inc., Chicago, IL, USA, 2012). The data was analyzed with the Mann–Whitney U-test. The data were expressed as means ± standard deviation (SD). Differences were considered significant at *p* < 0.05 and highly significant at *p* < 0.01.

## 5. Conclusions

CXCR4 inhibition with plerixafor (AMD3100) did not affect spheroid formation in Ewing’s sarcoma cells under 24 h simulated microgravity. Spheroidal adhesion occurred under altered expression of genes associated with malignancy. Especially *EWS/FLI1*, *CXCR4*, and *CD44* expression levels increased considerably, while corresponding protein levels were generally less affected. Hyaluronic acid receptor CD44 protein level significantly decreased contrary to an increased gene expression. Only in adherent cells increased EWS/FLI1 expression led to increased protein levels. Further characterization of the underlying mechanisms could improve knowledge about three-dimensional cell cluster formation and adherence, triggers for changes in cellular adhesion patterns and three-dimensional ES tumor biology.

## Figures and Tables

**Table 1:** This table shows gene expression alterations of genes tested after 24 h of s-µg. The comparative CT (ΔΔCT) method was used for relative quantification of transcription levels, with the control group set as 100%. Each value is displayed as the x-fold of the corresponding expression level of the control group. Upward pointing arrow (↑) symbolizes increase in gene expression and downward pointing arrow (↓) symbolizes decreased gene expression of the respective gene, equal sign (=) means no changed gene expression (values between 0.9x-1.1x). Values marked with an asterisk have shown to be significant (*p* ≤ 0.05)

Gene	Control (Set as 1)	Spheroid Under s-µg		Adherent Under s-µg	
*EWS/FLI1*	-	18.5x *	↑	8.2x *	↑
*CXCR4*	-	27.2x *	↑	0.9x	**=**
*CD 44*	-	1.5x	↑	3.7x *	↑
*CAV 1*	-	1.2x *	↑	1.3x *	↑
*VEGF-A*	-	0.9x	**=**	0.7x *	↓
*DKK2*	-	0.8x *	↓	0.9x	**=**
*LOX*	-	0.6x	↓	0.6x	↓
*MMP9*	-	0.9x	**=**	1.0x	**=**
*ERBB4*	-	0.8x	↓	0.9x	**=**
*NKX2.2*	-	1.2x	↑	0.9x	**=**
*CD99*	-	1.0x	**=**	1.0x	**=**
*ZEB2*	-	1.0x	**=**	1.0x	**=**
*CXCR7*	-	1.0x	**=**	1.1x	**=**
*IGFR1*	-	0.8x	↓	0.8x	↓

* significant (*p* ≤ 0.05).

**Table 2:** This table shows the primer sequences used in this study. All genes that are listed with a citation are not only verified via Primer Blast software but also used in another paper.

Gene	Sequences
*EWS/FLI1* [119]	fwd 5’ GCACCTCCATCCTACCCTCCT 3’ rev 5’ TGGCAGTGGGTGGGTCTTCAT 3’
*LOX*	fwd 5’ GGCGACGACCCTTACAACC 3’ rev 5’ CTGGGAGACCGTACTGGAAGT 3’
*CD44* [24]	fwd 5’ ACCCTCCCCTCATTCACCAT 3’ rev 5’ GTTGTACTACTAGGAGTTGCCTGGATT 3’
*MMP9* [120]	fwd 5’ CGCGCTGGGCTTAGATCATT 3’ rev 5’ GGGCGAGGACCATAGAGGT 3’
*ZEB2* [120]	fwd 5’ AAGCCAGGGACAGATCAGC 3’ rev 5’ CCACACTCTGTGCATTTGAACT 3’
*NKX.2.2*	fwd 5’ GCCCGAGCCAGCCAAGAGG 3’ rev 5’ GCCAGACCGTGCAGGGAGTA 3’
*ERBB 4* [121]	fwd 5’ TGTGAGAAGATGGAAGATGGC 3’ rev 5’ GTTGTGGTAAAGTGGAATGGC 3’
*CAV* 1 [24]	fwd 5’ GTACGACGCGCACACCAA 3’ rev 5’ TCCCTTCTGGTTCTGCAATCA 3’
*VEGF-A* [24]	fwd 5’ GCGCTGATAGACATCCATGAAC 3’ rev 5’ CTACCTCCACCATGCCAAGTG 3’
*E-Cadherin*	fwd 5’ CCCGGGACAACGTTTATTAC 3’ rev 5’ GCTGGCTCAAGTCAAAGTCC 3’
*CD99* [122]	fwd 5’ TAGGAGATGCTGTTGTTGATGGA 3’ rev 5’ GGATTTGGCATCGGTTTGG 3’
*CXCR 4*	fwd 5’ CACTCCCGCCCAATATACCC 3’ rev 5’ TCTGAAGTGTATATCATTCTGGGCT 3’
*CXCR 7*	fwd 5’ CAGCAGAGCTCACAGTTGTTG 3’ rev 5’ CGGGCAATCAAATGACCTCC 3’
*IGF1-R* [123]	fwd 5’ CCATTCTCATGCCTTGGTCT 3’ rev 5’ TGCAAGTTCTGGTTGTCGAG 3’
*HPRT1* (Housekeeper) [118]	fwd 5’ TGACCTTGATTTATTTTGCATACC 3’ rev 5’ CGAGCAAGACGTTCAGTCCT 3’

## Supplementary Materials

Supplementary materials can be found at https://www.mdpi.com/1422-0067/20/23/6073/s1.

## Abbreviations

1g	gravitational force equivalent of 1
2D	two-dimensional
3D	three-dimensional
DAPI	4′,6-diamidine-2-phenylindol
DNA	Deoxyribonucleic acid
cDNA	complementary DNA
DPBS	Dulbecco’s phosphate-buffered saline
ECM	Extracellular matrix
e.g.,	Exempli gratia
ES	Ewing’s sarcoma
FCS	fetal calf serum
kD	Kilodalton
MCS	Multicellular spheroids
MW	molecular weight
PBS	phosphate-buffered saline
r-µg	Real microgravity
s-µg	Simulated microgravity
RNA	ribonucleic acid
RBP	RNA-binding protein
mRNA	messenger ribonucleic acid
miRNA	micro RNA
RPM	Random Positioning Machine
rpm	rounds per min
UPR	unfolded protein response
