# Supplementary material for: The Role of C-X-C Chemokine Receptor Type 4 (CXCR4) in Cell Adherence and Spheroid Formation of Human Ewing’s Sarcoma Cells under Simulated Microgravity
Source: Int J Mol Sci. 2019 Dec 2;20(23):6073. doi: 10.3390/ijms20236073 (PMC6929163; doi:10.3390/ijms20236073)
Supplement: Supplementary file 1 [file ijms-20-06073-s001.pdf]

Supplementary Material

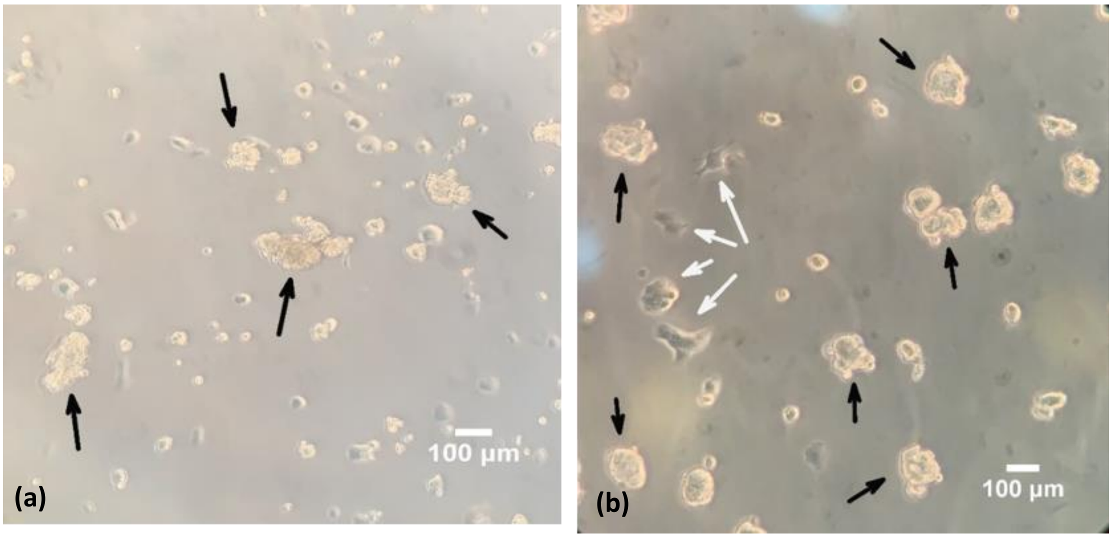

Supplementary Figure 1. (**a**) (**b**) various spheroids (black arrows) after 24h under simulated microgravity. On the ground of the cell culture flask some adherent cells (white arrows) are still attached to the culture flask surface. (Not every spheroid or adherent cell is highlighted).

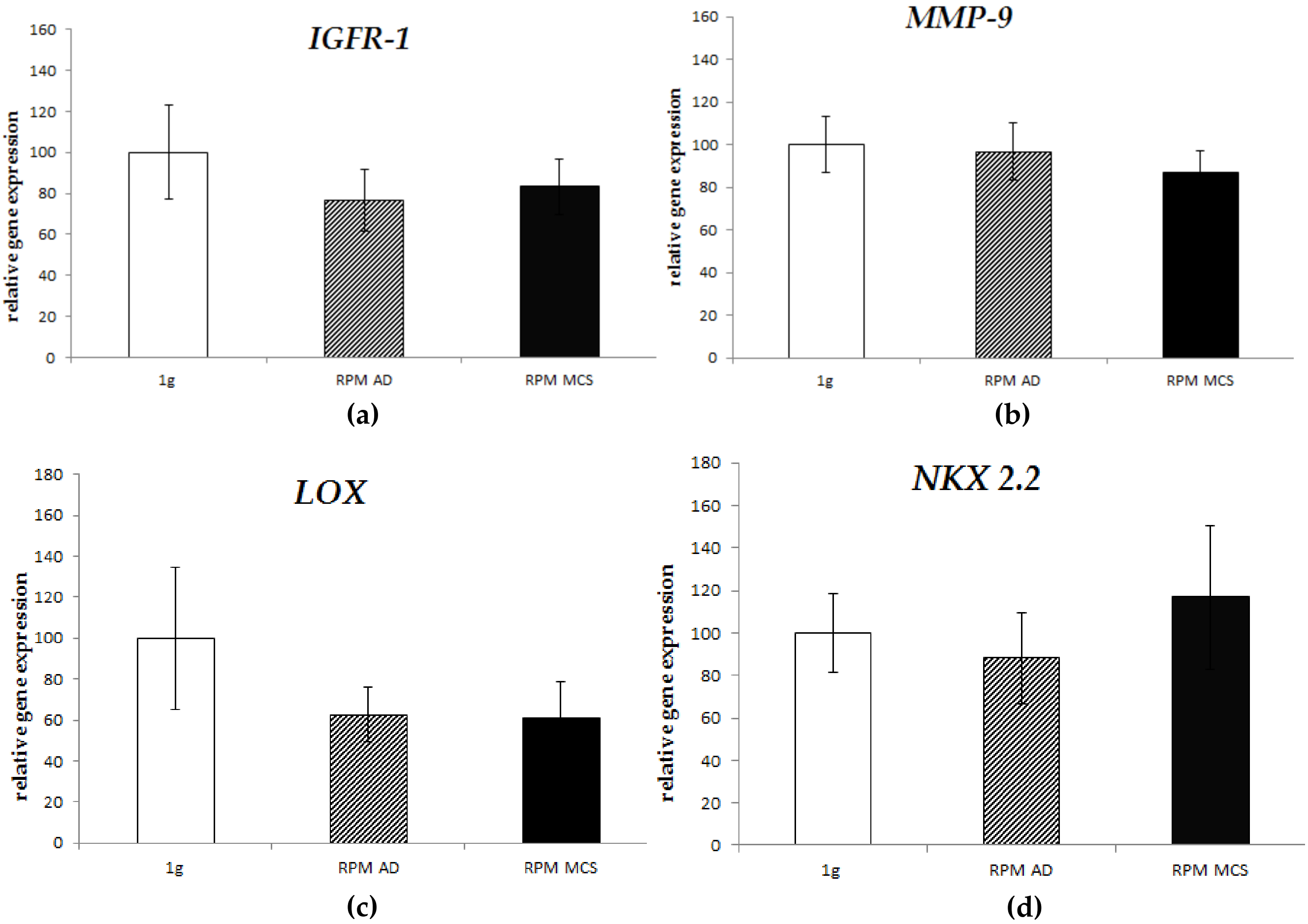

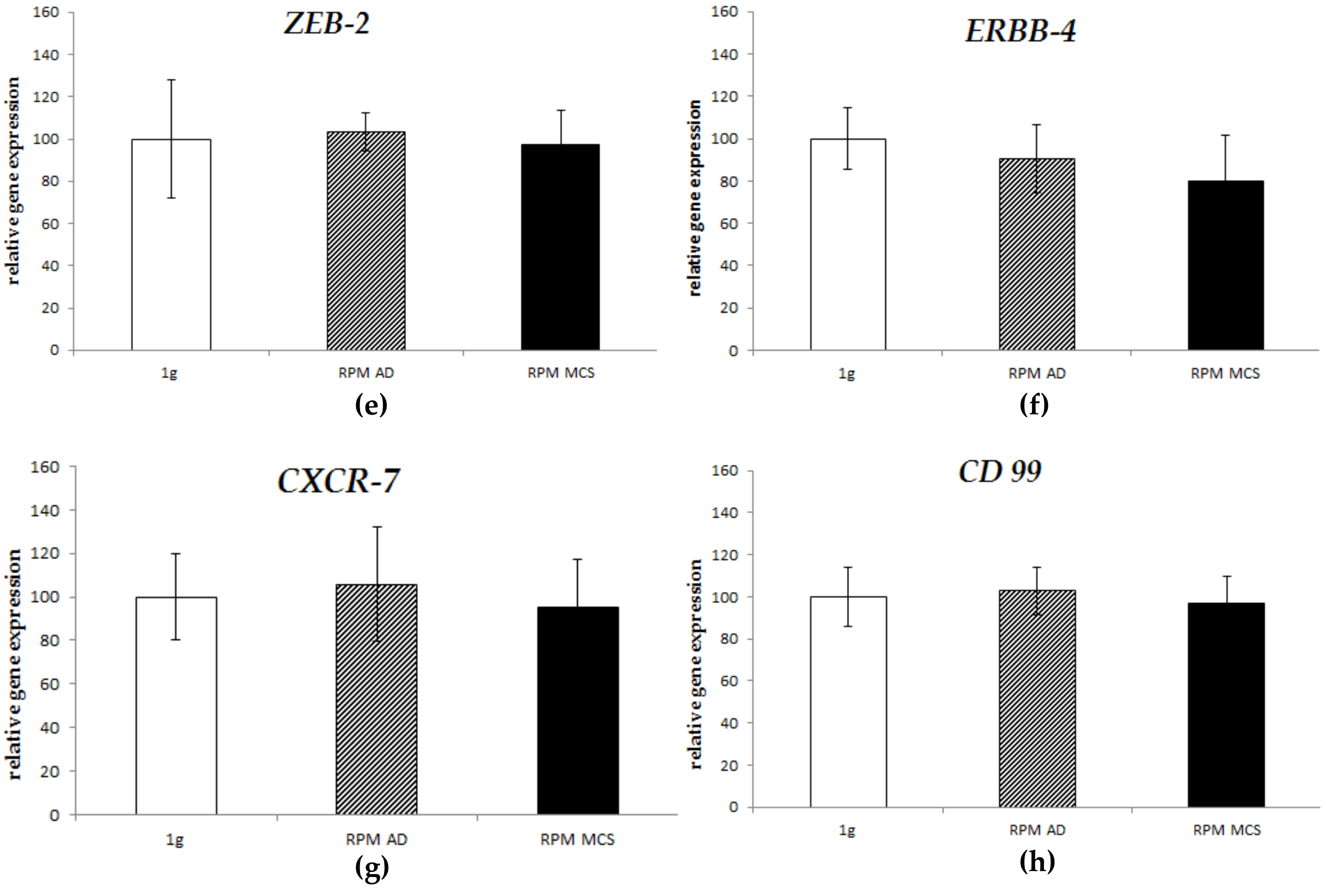

Supplementary Figure 2. Figures 2 (a) – (h) show relative gene expressions compared to 1g-control group. None of the gene shown reached significance.
